# Identification of transposable element families from pangenome polymorphisms

**DOI:** 10.1186/s13100-024-00323-y

**Published:** 2024-06-26

**Authors:** Pío Sierra, Richard Durbin

**Affiliations:** Department of Genetics, University of Cambridge, Cambridge, CB2 3EH UK

**Keywords:** Pangenome, Transposable element identification, Transposable element, Library creation, Insertion polymorphism

## Abstract

**Background:**

Transposable Elements (TEs) are segments of DNA, typically a few hundred base pairs up to several tens of thousands bases long, that have the ability to generate new copies of themselves in the genome. Most existing methods used to identify TEs in a newly sequenced genome are based on their repetitive character, together with detection based on homology and structural features. As new high quality assemblies become more common, including the availability of multiple independent assemblies from the same species, an alternative strategy for identification of TE families becomes possible in which we focus on the polymorphism at insertion sites caused by TE mobility.

**Results:**

We develop the idea of using the structural polymorphisms found in pangenomes to create a library of the TE families recently active in a species, or in a closely related group of species. We present a tool, pantera, that achieves this task, and illustrate its use both on species with well-curated libraries, and on new assemblies.

**Conclusions:**

Our results show that pantera is sensitive and accurate, tending to correctly identify complete elements with precise boundaries, and is particularly well suited to detect larger, low copy number TEs that are often undetected with existing de novo methods.

**Supplementary Information:**

The online version contains supplementary material available at 10.1186/s13100-024-00323-y.

## Background

Transposable Elements (TEs) often take up a large fraction of a eukaryotic genome, and they can also vary enormously between species [43]. Due to their repetitive nature, TEs represent a problem for accurate genome assembly and read mapping, in particular when dealing with short reads. For that reason they have frequently been disregarded in many analyses, typically by masking them out [39]. However, with the new longer read assembly protocols [23] it is now much easier to sequence through them so they are correctly represented in genome assemblies, making it easier to identify and classify them, and consequently to study them for their own sake.

The construction of a library of transposable elements present in an organism typically involves the following steps: search for elements homologous to those of an existing library for a closely related species; search for repetitive elements in a genome; search within these repetitive elements for defining structural features, such as LTRs (Long Terminal Repeats), TIRs (Terminal Inverted Repeats), known motifs and ORFs (Open Reading Frames) for characteristic proteins [41]. TE Hub [7] currently lists 51 tools associated with “library generation”. Two of the most popular ones are RepeatModeler [9] and REPET [8]. There are also composite methods that merge the results of multiple other tools in pipelines, such as EarlGrey [2], PiRATE [3], TransposonUltimate [38] and MCHelper [31]. It is also possible to process several genomes serially and combine their annotations to produce a more complete library [32]. However essentially all these tools carry out their search starting from the set of chromosomal or contig sequences of a single assembled genome, or the raw sequencing reads for a single genome [30]. In general these methods are characterised by a “repeat first” approach, in that they first look for dispersed repetitive sequences or structural motifs and then try to cluster them and extend them to recreate finally the complete sequence of the original TE. Here we instead take a “polymorphism first” approach, focusing on insertion polymorphisms between different copies of the genome as likely candidates for full length TEs, as described below.

Regardless of the method employed, to obtain a high quality library usually requires manual curation of the results, to ensure that the consensus sequences belong to TEs and not to other repeat types such as simple repeats or repeated gene families, and also to confirm that they represent the complete element, rather than just a fragment of it or an extension of it containing adjacent non-TE sequence [13]. Naturally, the extent to which the initial detection tool itself automatically creates full length consensus sequences for candidate TE families is very important in determining the amount of effort required to curate the library.

One of the changes that makes it possible to look with a new light at TE biology is the existence of large scale projects like the Darwin Tree of Life [42] or Zoonomia [11], which are rapidly increasing the number of species for which one or more high quality assemblies are available. The latest assemblers, like HiFiAsm [5] or VERKKO [36], aim to independently assemble the two haplotypes of a diploid genome. This provides multiple copies of genome sequences from the species that can be compared to identify structural polymorphisms at several levels. First, we can compare the two haplotypes of the same sample (or more if it is polyploid) to obtain heterozygous polymorphisms. Second, we can compare the genomes from two or more individuals of the same species to obtain intraspecies polymorphisms. Lastly, we can compare the genomes of two or more closely related species with a high level of synteny to obtain interspecies polymorphisms.

The other important change is the appearance of faster and more accurate whole genome alignment tools [22, 27]. In particular methods have been developed to generate pangenomes which represent multiple closely related genome sequences in a computational graph structure which explicitly identifies sequence segments present only in a subset of the genomes, i.e. structural variants (SVs) [10, 15, 24]. Projects such as the Human Pangenome Reference Consortium [25] are generating large pangenome graphs of this nature. A new tool, GraffiTE [14], has been developed to use these pangenomes to genotype transposable element insertion polymorphisms, starting from an existing TE library for the species under consideration. In our work we follow the opposite direction to show how a pangenome can be used to obtain a new TE library whose elements will be close in most cases to the complete sequence of the TE, even before any manual curation. Recently a related method has been made available, which starts from short read whole genome shotgun data to identify polymorphic structural variants as candidates for TEs [4], and the Pannagram genome comparison software has also been used to find new TE families in a similar fashion [17].

## Methods

**Fig. 1:**
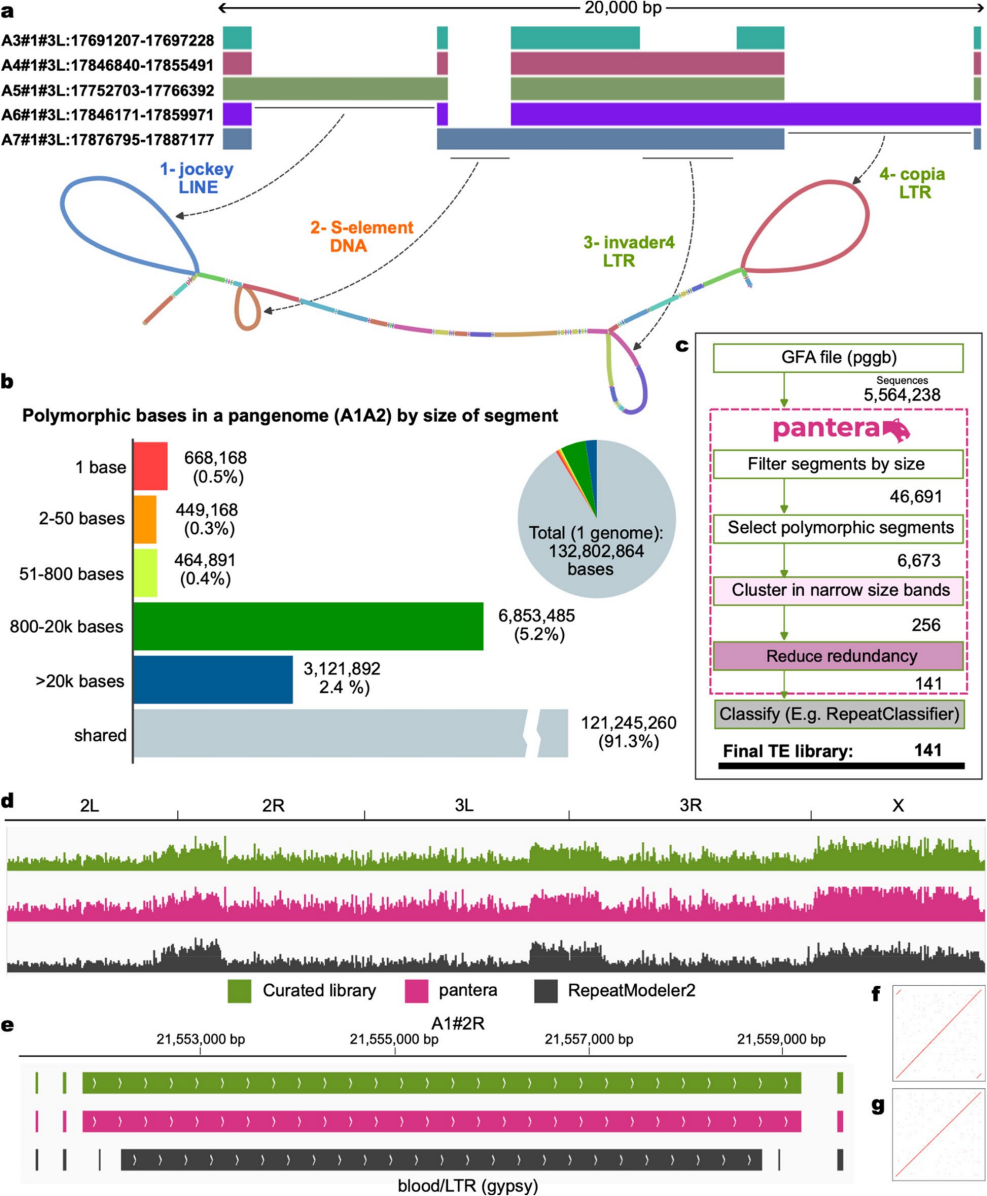
Obtaining a transposable element library from a pangenome. **a** A 20 kb section of a pangenome of chromosome 2R from seven high quality genomes of *Drosophila melanogaster* (just five shown, figure generated with odgi). Gaps in the bands represent structural variants, i.e. insertions or deletions in some of the genomes compared to the others. These structural variants can also be visualised as loops or “bubbles” in the graph representation. Here we see four structural variants each thousands of bases long, arising from four different TE insertions. **b** Number of bases in a pangenome of two *Drosophila melanogaster* genomes (A1 and A2) by whether the bases are fully aligned (shared) or they do not align, binned by the size of the insertion or mismatch. **c** Workflow of pantera**.** First it selects from the GFA file segments that are polymorphic and may hence belong to a TE. To reduce the number of false positives only segments for which there are at least two almost identical polymorphic sequences are selected (cluster in narrow size bands). Then, a less stringent clustering is performed to reduce redundancy and generate the final TE library that can be classified with any existing tools. **d** Annotations of the A1 *Drosophila melanogaster* genome obtained with RepeatMasker using three different libraries. Green: curated reference library. Pink: pantera de novo library. Grey: RepeatModeler de novo library. **e** Example of an LTR element (Blood) for which pantera was able to correctly identify the full element, including its LTR components (**f**) that in this case are not fully reported by RepeatModeler, neither as part of the full consensus (**g**) nor as a solo LTR element

Our hypothesis is that most of the SVs in the size range of hundreds to tens of thousands of bases that we detect when comparing two or more closely related genomes are caused by TE insertions or deletions, as illustrated in Fig. 1a,b. We identify candidate TE families by clustering the inserted sequences with high stringency for both sequence identity (default 95%) and full length alignment. Our tool returns consensus sequences of these putative TE families. These can later be classified into any of the more than 500 current superfamilies described in the literature using an existing classifier, in our case RepeatClassifier [9]. They can also be used directly with tools to annotate or mask TE copies in a genome, such as RepeatMasker [9]. Figure 1c provides an overview of the process, with Fig. 1d illustrating the resulting annotation created by RepeatMasker for the *Drosophila melanogaster* genome, and Fig. 1e,f,g an example of a specific identified element. Source code is available at https://github.com/piosierra/pantera.

### Prerequisites

The only required input to run pantera is a pangenome in the form of one or several GFA files, that include the individual paths on the graph as P entries or the links as L entries, all with non overlapping links between segments. We have generated these files with pggb [10], but other pangenome graph generators should also be usable. For the pangenomes described in this paper we used pggb parameters -p 85 to 95 (minimum average nucleotide identity for a seed mapping) and -s 2000 to 5000 (segment length), depending on the expected levels of identity of the sequences included, and workflow versions wfmash v0.9.1–3-gc5882a1 “Mutamento”, seqwish v0.7.6 “Temporaneo”, odgi v0.7.3 “Fissaggio”. No master reference is needed. When the pangenome is divided into several GFA files, for example one for each chromosome, pantera can take a list of GFA files to process.

### Segment selection and clustering

The first step is to select just the segments in the graph that belong to insertion/deletion polymorphisms. In the case of a graph made from just two genomes, such as the haplotypes of a diploid genome, we directly select segments that belong to just one path and are flanked by segments shared by both paths that are contiguous in the second path. To avoid an artefact of pangenomes generated by pggb in divergent regions we require that the flanking shared segments are at least 2 bp long. When the graph contains more than two paths, we apply this rule to each pair of paths. Next we filter these segments to select only those falling in a certain length range (defaults 250 to 50,000 bases).

Once the segments are filtered they are ordered by length and allocated into an initial set of bins based on overlapping length windows of width 200 bp. Any of these bins that contain more than 300 sequences is split so that the final bins contain no more than 300 sequences (for example a bin of size 800 is split into bins of 266, 267, 267). These bins are then clustered using an R implementation of the cd-hit algorithm [28] at default 95% identity (cd-hit-est options: -c 0.95 -G 1 -g 1). The bin size limit of 300 is an empirical threshold that provides enough examples to form a good consensus, while improving specificity where there are closely related families of similar sizes, and maintaining compute efficiency.

Sequences from clusters with at least a given number of sequences (default = 2) are then selected and any of the redundancy from the overlapping windows removed. The requirement to have the same sequence polymorphic in at least two different places in the genome effectively distinguishes transposable elements with a mechanism to copy a specific sequence between precise endpoints from other types of deletion or insertion that remove or insert arbitrary sequence.

Finally, these candidate TE clusters are clustered once again, with wider windows of width 2,000 bp, and the resulting “super-clusters” are aligned with mafft. A consensus is then obtained from the aligned sequences, using a stringent plurality threshold of 60% on the first pass that helps remove random edge sequences (Supplementary Fig. 1).

After producing the initial library we remove subfragments that are contained within complete TEs, such as solo LTRs or partial LINE elements. This is done by repeating the clustering process, reducing the identity needed to cluster (default on second pass 80%) but increasing the requirements of coverage for the smaller sequence aligned (cd-hit-est options: -c 0.85 -G 0 -aS 0.90 -uL 0.05 -g 1).

### TE family classification

The previous steps produce a set of sequences which are expected to belong to transposable element families. They can then be classified using any TE classifier tool; for example we used RepeatClassifier [9].

We also confirmed the classifications for a few of the families mentioned in the text using CENSOR [19] via the Repbase website to identify related previously classified families. In no case did this change our classification from RepeatClassifier.

### Assessment of completeness

Elements were defined as “complete” as follows: 1) for DNA elements (except Cryptons) and LTR DIRS elements, having terminal inverted repeat (TIR) sequences at least 10 bases long; 2) for LTR elements, having long terminal repeat (LTR) sequences at least 200 bases long; 3) for LINE elements, having a candidate ORF at least 1,300 aminoacids long, and a polyA tail. In addition LINE1 elements required a further ORF1 candidate at least 700 aminoacids long. As RepeatModeler returns results for LTR elements as two components, internal and LTR, when scoring RepeatModeler results we count as “complete” any LTR result with an internal segment larger than 3,000 bases that also has the corresponding LTR segment. The remainder of the LTR results are considered as incomplete. These can include solo LTR segments for which a copy of the full LTR element is no longer present in the genome.

To identify the features required for this analysis, open reading frames (ORFs) were obtained with getorf [37] and structural features (LTRs, TIRs, polyA tail) detected with our own script pantercheck, in which TE candidate sequences are blasted to themselves one by one to find internal repeats, with default blastn parameters allowing one mismatch for each eight bases.

## Results

**Fig. 2:**
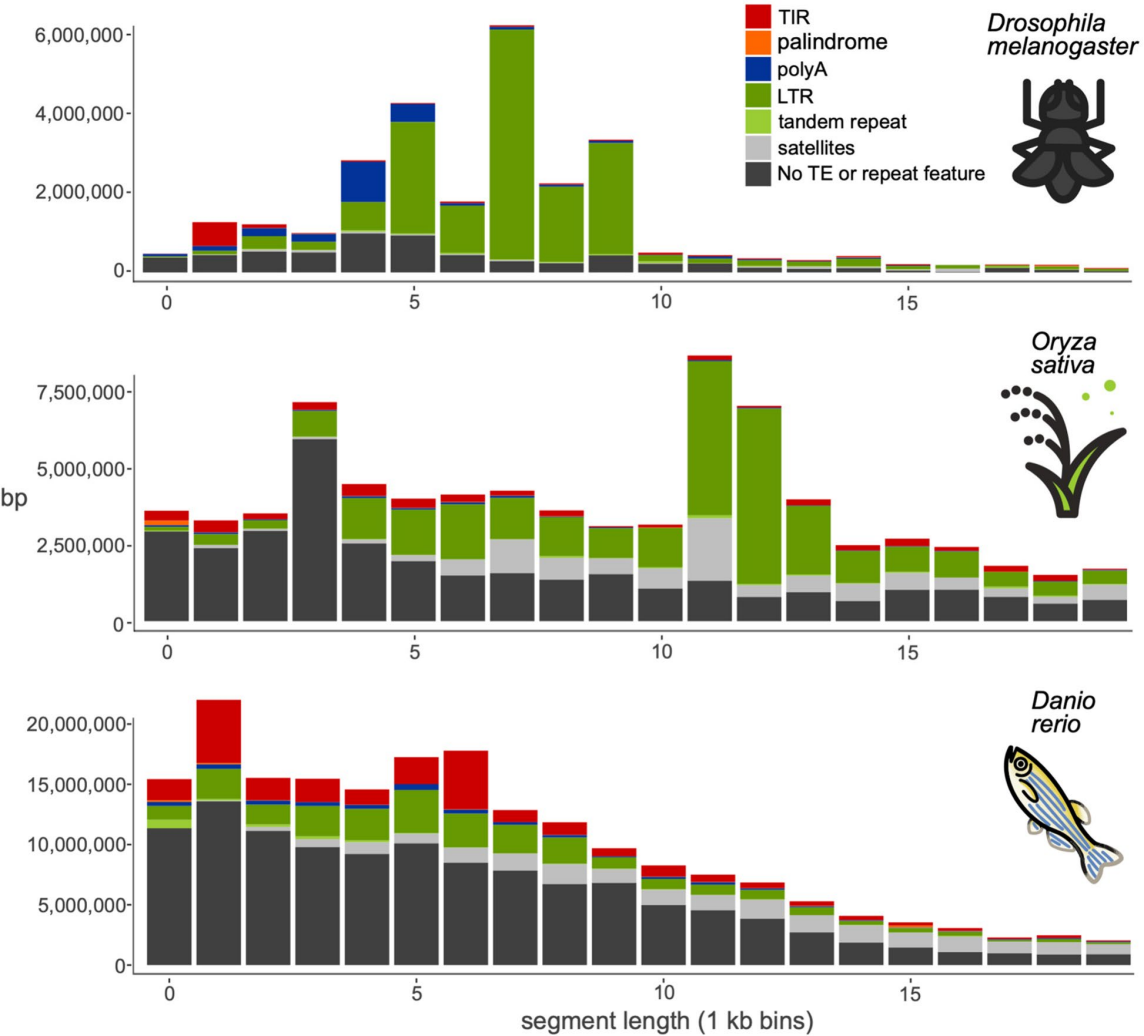
Structural feature found in polymorphic segments of three pangenomes, by segment length (250—20,000 bases). Total number of base pairs in insertions on a pangenome in different size ranges, grouped by structural features associated with TEs or other repeats. TIR: terminal inverted repeats. Palindrome: TIRs that occupy more than 90% of the sequence. polyA: A/T homopolymer at least 10 bases long, allowing for 1 mismatch every 8 bases. Tandem repeat: The sequence is composed of a smaller sequence repeated 2 or 3 times. Satellites: The sequence is composed of a motif repeated more than 3 times

First, we benchmarked the new tool with three species for which there exist manually curated TE libraries: *Drosophila melanogaster* (fruit fly)*, Oryza sativa* (rice), *Danio rerio* (zebrafish). Figure 2 shows the distribution of insertion polymorphisms in the size range 250 bp to 20 kb in the pangenomes we used for these species, with the fraction of those polymorphisms exhibiting the various TE features classified by pantercheck. For each of the species we compared the results of pantera to those of a reference library and those obtained with a denovo method, RepeatModeler for *Drosophila melanogaster* and *Danio rerio* and REPET for *Oryza sativa.*

### *Drosophila melanogaster*

We used seven genomes A1 to A7 of *Drosophila melanogaster* from the DrosOmics project [6] (Fig. 1a) and built a pangenome composed of one connected graph for each of the five main Muller elements (chromosome arms 2L, 2R, 3L, 3R and X). This produced 5 GFA files between 100 and 144 MB of size composed of a total of 5,564,238 segments. Running pantera on them resulted in a library of 141 elements that we classified using RepeatClassifier 2.0.4 [9] (Fig. 1c). Next we ran RepeatModeler 2.0.4 [9] with default parameters on one of the genomes (A1) and compared the results (N = 361) with pantera and the reference library, Drosophila transposon canonical sequences (v10.2), obtained from https://github.com/bergmanlab/drosophila-transposons.

### *Oryza sativa*

For rice we constructed the pangenome from two genome sequences: the reference genome GCF_001433935.1 from the Japonica group [18] and an Indica group genome GCA_001623345.3 [44]. The final graph was composed of 7,801,181 segments. The resulting library obtained with pantera had 525 elements of which 267 (51%) are classified by RepeatClassifier as Unknown. We compared this library to the manually curated TE annotation in Rice (v6.9.5) [33], with 2,431 elements. In this case we compared the results of pantera to the uncurated library obtained with REPET [8, 34, 35] downloaded directly from REPETDB [1] composed of 2,479 families.

### *Danio rerio*

For zebrafish we used the reference genome danRer11 (GCF_000002035.6) [16] and compared it to fDanRer4.1 (GCA_944039275.1), one of the recent assemblies generated by the Wellcome Sanger Institute Tree of Life programme. The final graph was composed of 32,943,885 segments. The library obtained from it using pantera returned 913 putative TE families with 29 (3%) of them being classified as unknown. We compared it to the 1,740 curated TE families included in Dfam [40], and to the results obtained with RepeatModeler2 (3,728 families).

### Benchmark results

**Fig. 3:**
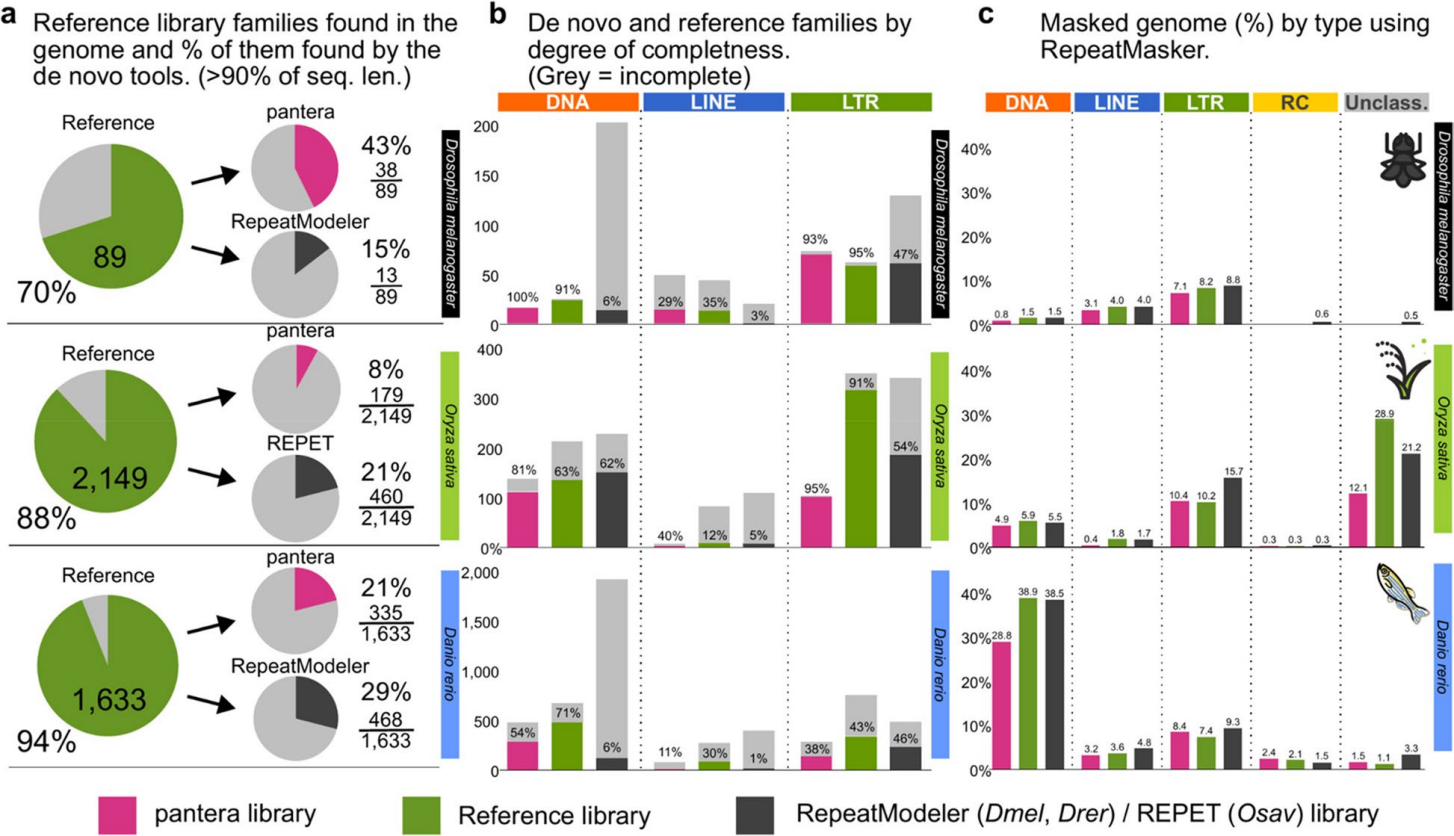
Comparing different TE libraries in *Drosophila melanogaster, Oryza sativa and Danio rerio*. **a** Number of families from the reference library with matches in the specific target genome (A1 for Drosophila, the standard reference for rice and zebrafish), and how many of them have a match with the Pantera or alternate automated library covering > 90% identity and length in the selected genome. Note that not all reference library families were found in the specific genome used (some are only found in other genomes from the species). **b** Degree of TE completeness as percentages of the total number of segments for each tool, type and species. The definition of “complete” is given in Methods subsection “Assessment of completeness". **c** Percentage of the genome masked by RepeatMasker using each of the libraries by type of TE family

To compare the results we looked at different values (Fig. 3): a) how many sequences from the reference library had at least 90% of their sequence matched by a sequence of the other tools; b) what fraction of the sequences obtained for each type were complete; c) the total percentage of the genome masked by the resulting libraries.

Pantera found more near-full length (> 90%) members of the reference libraries than RepeatModeler (fruit fly and zebrafish) or REPET (rice) except for LTR elements for rice and zebrafish. In rice this was primarily due to different criteria on divergence while defining a family, as was confirmed by the similar percentage of genome masked in both cases (pantera 10.4%, reference 10.2%). In the case of zebrafish both pantera and RepeatModeler libraries have an excess of incomplete elements, probably due to the relatively low copy number of full length LTR elements.

**Fig. 4:**
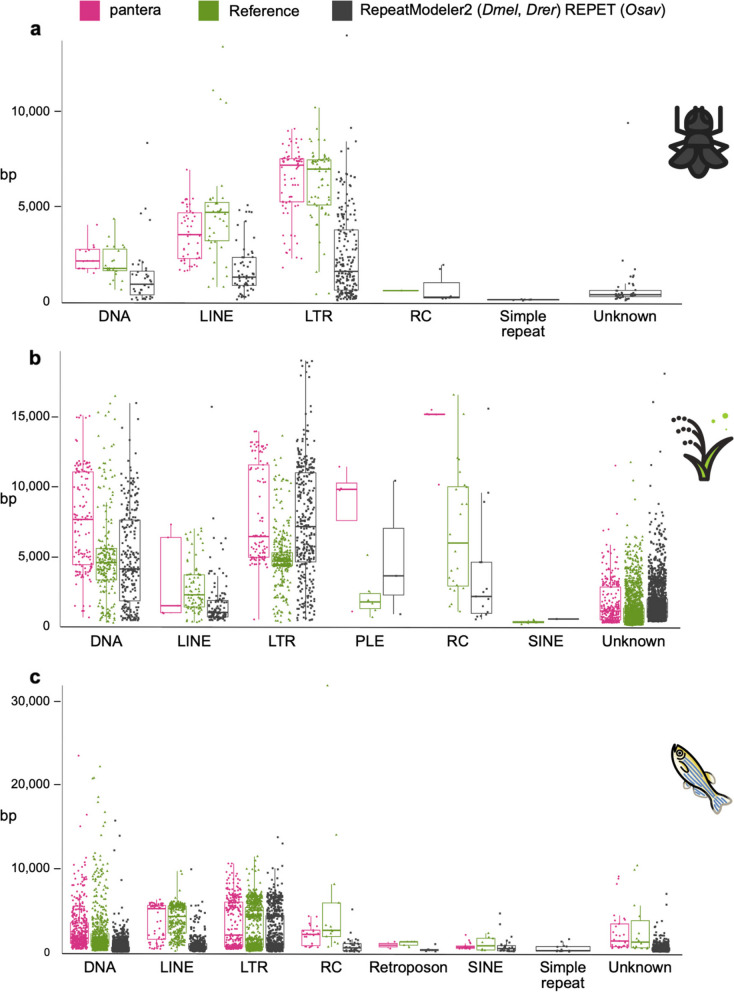
Length distributions of the different libraries by TE order. Length distributions of the consensus sequences by order in which they have been classified. RC stands for rolling circle (Helitrons). **a** *Drosophila melanogaster*. pantera (*N* = 141), Drosophila Transposon Canonical Sequences 10.2 (*N* = 127), RepeatModeler (*N* = 361). **b** *Oryza sativa*. pantera (*N* = 525), rice6.9.5 (*N* = 2431), REPET (*N* = 2471) **c** *Danio rerio*. pantera (*N* = 913), Dfam curated (*N* = 1740), RepeatModeler (*N* = 3728)

In general pantera families are more complete as defined in the previous section, even than the reference library families (Fig. 3b), with the exceptions being DNA and LTR elements for zebrafish. Length distributions of all families generated can be compared in Fig. 4 and Supplementary Fig. 2. We interpret the typically longer mean size and lower variance of the distributions of lengths by superfamilies for pantera as further evidence that the consensus sequences it produces tend to belong to full elements. As an example, of 48 CMC-EnSpm families identified by pantera, only 5 lack the expected TIR elements, compared to 29 families missing the TIR element out of 70 in the REPET results. This is even true for LINE elements, for which it is particularly hard to produce a full length consensus because most copies are incomplete. Another point to take into account is that the results can also be biased by the cut point selected to define the minimum size of an element to be included in the library. Mobile elements associated with TE activity usually start over the 100 bases mark, with SINEs or solo LTRs. If instead we want to focus on autonomous TEs, in our experience a minimum size of 700 to 800 bases is low enough. As pantera uses the information from several genomes, it is possible that a family found in the pangenome is not actually present in one of the genomes. This happens for example with the full Q-element, LINE/CR1, in fruit fly, that can be found in the curated and pantera libraries, but not present in the genome (A1) used by RepeatModeler and as template for the results.

The results of masking the genomes with the libraries generally show a comparable though slightly lower coverage percentage by pantera (Fig. 3c). This is expected as pantera will not build consensus sequences from very old and fragmented TE insertions, that can represent a sizable percentage of the genome, and instead will identify more recent elements, which are closer to the putative active sequence of the TE, but which may have fewer copies in the genome. As an example, in *Danio rerio* pantera identified one large CMC-EnSpm element that has three full copies in the genome. It shows the two full proteins associated with these elements, and has a 13 basepair TIR (CACTCAAAAAAAT) (Supplementary Fig. 3). This and other large CMC (CACTA) elements were not reported by RepeatModeler. The same happened with other large DNA elements classified as Zisupton (Supplementary Fig. 4).

RepeatMasker landscape plots for all libraries are shown in Supplementary Figures 5,6 and 7. Differences between methods are observed due to different clustering approaches. In general pantera provides greater resolution at low Kimura divergences, presumably due to its tight initial clustering step.

We compared the time employed by all workflows (Supplementary Fig. 8) except for the REPET library for rice for which we used a library previously generated. For the pantera workflow we added the time employed by the creation of the pangenome (pggb) extraction of the library (pantera) and the classification of the sequences (RepeatClassifier). The results for RepeatModeler include also the time employed by RepeatClassifier. With *Drosophila melanogaster* pantera was 3.5 × times faster than RepeatModeler, even though the pangenome was composed of 7 genomes. In the case of *Danio rerio* the pantera workflow was 6 × times faster than RepeatModeler. It is worth noting that by default RepeatModeler limits the genome sampled to 400 MB. This limit can be increased to sample the full genome and avoid missing low copy elements, but that comes at a larger cost in execution time. The results presented used the default configuration.

### Results using both haplotypes of the same sample: *Trachurustrachurus* and *Aquila chrysaetos*

**Fig. 5:**
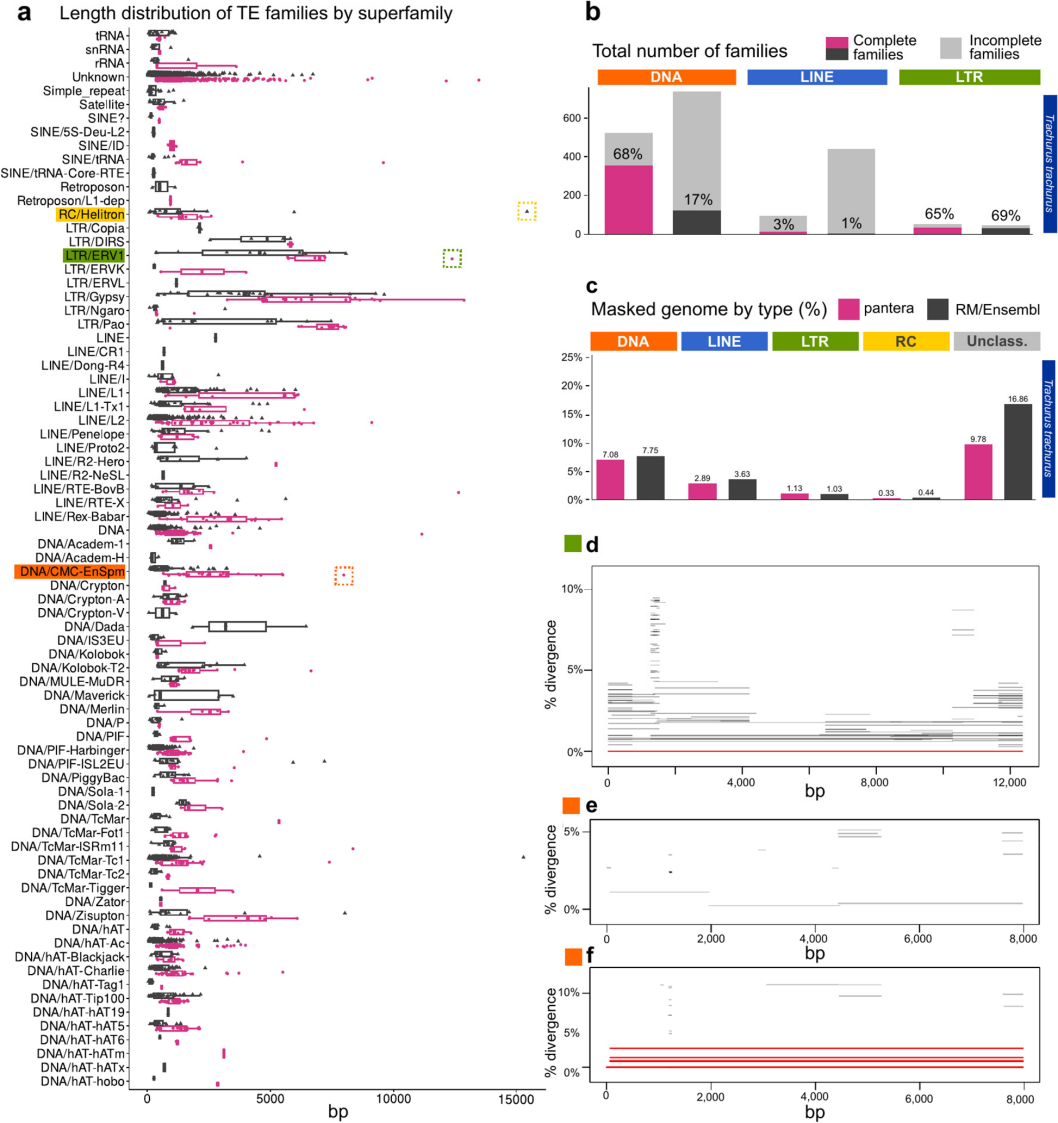
Results with *Trachurus trachurus*, from a pangenome composed from the primary and alternate assemblies from the same sample. **a** Length distributions of the consensus sequences by superfamily in which they have been classified. pantera (*N* = 1301), RepeatModeler (*N* = 3718). Highlighted in dotted boxes are a Helitron element not found by pantera (yellow), and an ERV1 (green) and a CMC-EnSpm (orange) element not found by RepeatModeler. **b** Total number of families of the resulting libraries, and their degree of completeness as in Fig. 2. **c** Percentage of the genome masked by RepeatMasker using each of the libraries. **d** Only one full length copy of the ERV1 boxed in green in **(a)** is present in the primary assembly. **e,** No full length copies of the CACTA element highlighted in orange in (a) are present in the primary assembly, while (**f)** six are present in the alternate assembly.** d**, **e** and **f** were generated with TE-aid (https://github.com/clemgoub/TE-Aid). [13]

As an example of the application of pantera to a newly sequenced species without a reference we selected two species from the Sanger Institute, the Atlantic horse mackerel *Trachurus trachurus* and the golden eagle *Aquila chrysaetos*. For *T. trachurus* we used its primary (GCA_905171665) and alternate (GCA_905171655) haplotype assemblies [12] to extract a new TE library for the species using the pantera pipeline (1301 families). Then we compared the results with the Ensembl annotation for the species, obtained with RepeatModeler, without further manual curation (3718 families) (Fig. 5). The results of masking the genome with both libraries are similar, but in the case of pantera more than double the elements in all three main divisions (DNA, LINE, LTR) appear to represent the full sequence of the TE. Of the DNA TEs found by RepeatModeler 29% had a more complete element in pantera. For LTRs the corresponding figure was 44%, but it was smaller for LINE elements, just 8%. As an example of a novel full length element, pantera identified a new ERV element, 12,371 bases long, with 707 bp LTRs and two ORFs of 1,402 and 1,032 amino acids (green box in Fig. 5a). There is just one full copy in the main haplotype and this is a case in which pantera can benefit from using the information present in both haplotypes (Fig. 5d). Furthermore, the largest family of CMC-EnSpm elements found in the genome has no full copies in the primary haplotype but is only present in the alternate haplotype with six full copies (Fig. 5e,f). In both of them we could observe the orfs encoding the full proteins characteristic of these families.

We repeated the same procedure with the primary haplotype (GCA_900496995.4) and alternate (GCA_902153765.2) of the golden eagle [29]. In this case pantera did not find any of the DNA type content found by RepeatModeler, as that appears to be due to old insertions which are no longer polymorphic. Instead, it was able to correctly find several large ERVs that are still polymorphic and might be relatively recent insertions, which were missed by RepeatModeler. In particular one of them includes an extra protein in addition to the putative ERV proteins that we found to be present also in the genomes of other Accipitriformes but not in more divergent species, which suggests that it could be an ERV specific to this order (Supplementary Fig. 9).

### Results comparing closely related species: *Astatotilapia calliptera* and *Maylandia zebra*

**Fig. 6:**
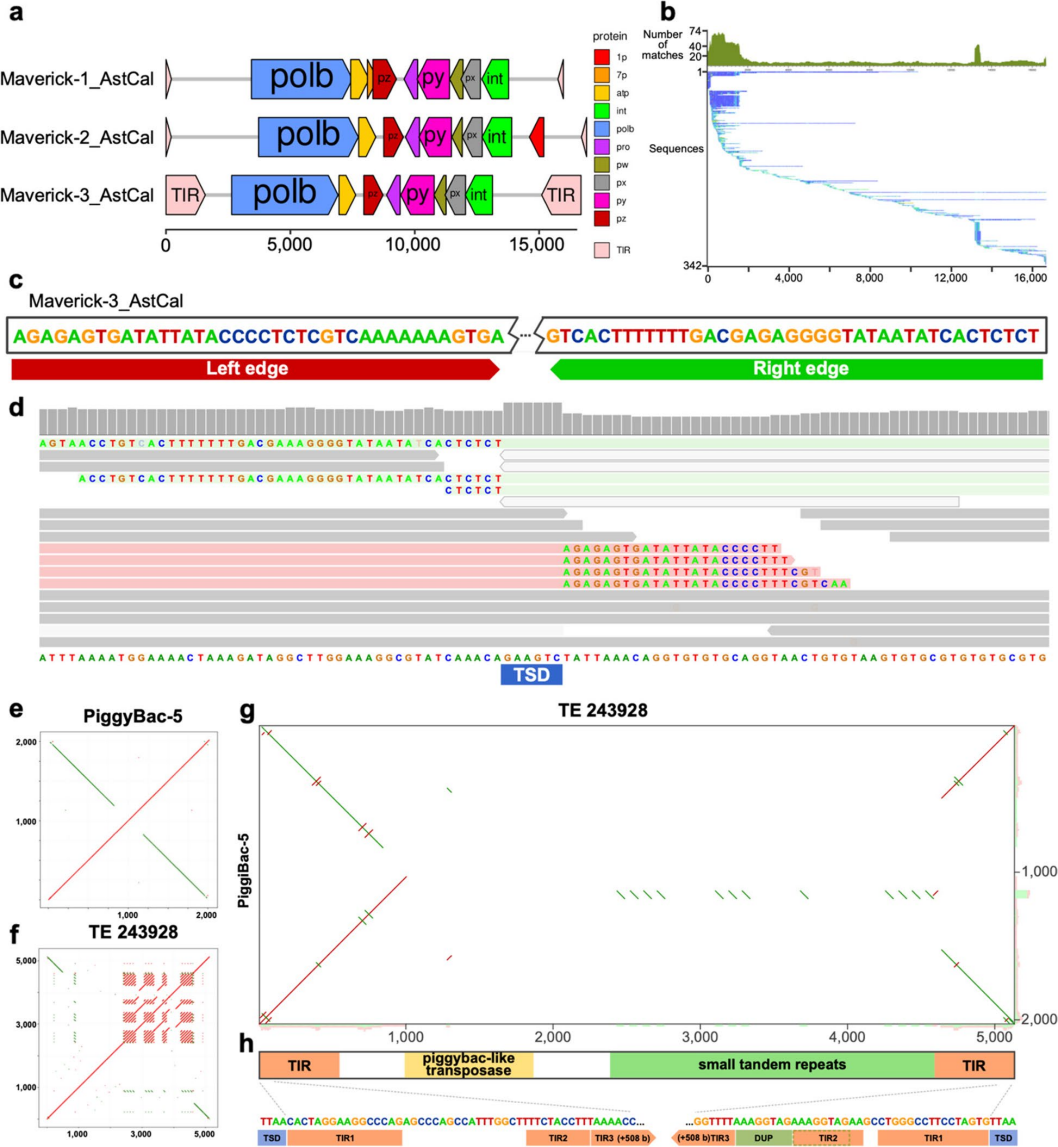
Selected TE families found in *Astatotilapia calliptera*. **a** Structure elements identified in three Maverick families found with pantera in a pangenome built with *Astatotilapia calliptera* and *Maylandia zebra*. All families include TIR elements, and separate ORF components for DNA polymerase b, integrase, ATPase and a double jelly roll capsid protein (py) among others. **b** Seed Alignment Coverage and Whisker Plot for Maverick-3_AstCal from Dfam (https://www.dfam.org/family/DF003572096/seed). The small number of matches all along its sequence can make it very hard to find based only on repetitiveness, but the presence of full elements in the Maylandia zebra genome allowed us to obtain the full sequence. **c** Detail of the edge sequences for both TIR elements. **d** The accurate definition of the edges of the TE element allows us later to use other tools like MeGANE to identify polymorphic insertions using short reads, bases on mapping of discordant reads to the TE sequence but also matching the soft clip reads of on the insertion to the edges of the putative TE element. In this case we observe the signal for an heterozygous polymorphic insertion, which has created an 8 bases target segment duplication (TSD). **e** Structure of a new TE composed of the fusion of two identical piggybac elements in opposite sense. **f** Hits in the genome. The black divergent lines show matches to previous insertions of the single piggybac element. The red complete ones prove that the new element is creating new copies. **g** Self dotplot showing the structure of the element. **f** and **g** generated with TE-aid (https://github.com/clemgoub/TE-Aid)

The polymorphism-first approach can also be applied to comparisons between genomes of closely related species, and we have found that in some cases this allows us to have a better understanding of their TE content. As an example, we created a pangenome from the genomes of two closely related cichlid fishes from Lake Malawi, *Astatotilapia calliptera* (GCA_900246225.5) and *Maylandia zebra* (GCA_000238955.5) that diverged within the last million years [26], and used pantera to generate 250 candidate TE families. In the resulting library we found three different complete families of Maverick elements for which previously only fragmented components had been reported. One of them, Maverick-3_AstCal, has just one full copy in the *Astatotilapia calliptera* reference genome (Fig. 6a,b), but a search for polymorphic insertions in more than 600 samples with short read data using MeGANE [20] confirmed that all of them have tens of polymorphic insertions of that family, highlighting the relevance of having the most complete possible consensus sequence to perform further downstream analysis accurately (Fig. 6c,d). Pantera also found a previously identified element, named piggybac-5, formed by the fusion of two segments of the same piggybac-like element in opposite senses (Fig. 6e,f,g). This has lost the transposase, but the intact TIRs suggest it is still being mobilized as a nonautonomous element, and indeed there are 51 full length copies in the *Astatotilapia calliptera* reference genome. Pantera also obtained a consensus for an intact piggybac TE (TE-243928) (Fig. 6f) which has only six full copies in the *Astatotilapia calliptera* genome, each containing a complete piggybac transposase of 256 aa. The target site duplications of TE-243928 and piggybac-5 are identical, and the terminal region of the TIR of piggybac-5 is the same as the TIR of TE-243928, but the piggybac-5 TIR is substantially extended internally by material which is only found in single copy in TE-243928 (Fig. 6h). We suggest that piggybac-5 may have been formed by overlapping chromosomal inversion events from TE-243928 or a closely related element.

## Discussion

The pros and cons of pantera are related to the “polymorphism first” approach. A benefit is that the consensus sequences obtained are often closer to the curated sequences than with standard “repeat first” approaches, when those sequences belong to recent elements in which the structural features are still intact. This is reflected also in obtaining a smaller percentage of sequences that could not be assigned to any category (Supplementary Tables 2–4). This is the result of the initial selection of segments (polymorphic and highly similar), that are more likely to be the product of recent transposition events and so reflect full TE elements. Unlike methods starting from a seed that is extended until the final consensus is found, our method is less constrained by size, as the size of the segments found is a direct result of the pangenome construction, and many of the segments originally selected are expected to represent the full sequence of the TE. Another benefit is that it does not need a large number of repetitive elements to identify the putative sequences, relying instead on the presence of at least two polymorphic copies in the pangenome.

We show several examples where pantera identified TE families missed by other automated methods (Figs. 5d-h, 6b). In particular, because it initially clusters full length sequences with high stringency, it can distinguish related separately transposing sequences that share common sections, illustrating how novel elements can arise from fragments of previous ones (Fig. 6e-h).

The tendency to generate full length consensus sequences is valuable for downstream tools which require an accurate library to genotype TE polymorphisms [42], particularly in species that contain hundreds or thousands of TE families, making it a daunting prospect to manually curate them. These tools to genotype TEs based on short reads usually are based on the information obtained from discordant read mapping to TEs and on the unmapped content of split reads. In both cases it is necessary to use the full TE consensus to accurately assign a polymorphism to that family (Fig. 6-c,d).

A limitation of the applicability of pantera is that, since it is a comparative method, it requires more than one genome sequence. Furthermore, elements which are not polymorphic between the genomes will not be identified. It might be expected that this would restrict pantera to only finding very recently transposed sequences, but as we saw in our evaluation it is surprisingly successful at generating a library that masks a similar fraction of the genome as more traditional approaches. It seems that for old insertions this is typically achieved not by including a consensus for the original TE in the library, but rather by masking with a more recently active descendant (or other close relative). We note that such a relative is normally no more divergent from the original TE than the actual insertions themselves—both will have drifted by mutation since their shared common ancestor. However, we recognise that ideally for old insertions it would be preferable to use a consensus that could in principle have only half the divergence from each instance of that of a recently active descendant reconstructed by pantera.

For this and other reasons, we do not claim that pantera by itself will replace existing approaches. Instead we suggest that it has complementary properties to them and will provide a valuable addition to composite TE annotation approaches such as EarlGrey alongside repeat-first methods. Because pantera tends to generate full length consensus sequences more frequently than tools that start from a repeat-first approach, we suggest that it might be used first, then the genome be masked for sequences found by pantera, then a method such as RepeatModeler or REPET be used on the masked genome.

## Conclusions

We present a novel approach to the identification of TEs based on insertion polymorphism, together with a practical software implementation, pantera. The results of this approach are complementary to those of previous automated TE family discovery tools and can be used to reduce the curation required to build a high quality TE library.

To make libraries for a new species it relies on there being multiple assemblies of a genome from different haplotypes, but these are now standard from modern long-read genome assemblies as generated for example by the Vertebrate Genomes Project [43], the Darwin Tree of Life project [15] or other Earth Biogenome Project [21] sequencing projects. As more species have their genomes sequenced it will be increasingly possible to apply pantera between closely related species.

### Supplementary Information

Supplementary Material 1.

Supplementary Material 2.

## Data Availability

The code for pantera and all the libraries generated by it that are described in this paper are available from https://github.com/piosierra/pantera. *D. melanogaster* genome sequences from DrosOmics can be downloaded from https://www.biologiaevolutiva.org/gonzalez_lab/drosomics/DATA/. Accession numbers for the *O. sativa* and *D. rerio* genome sequences used are given in the main text.

## Abbreviations

GFA: Graphical fragmented assembly format
LTR: Long terminal repeat
SV: Structural variant
TE: Transposable element
TIR: Terminal inverted repeat
TSD: Target site duplication
