# Supplementary material for: Identification of transposable element families from pangenome polymorphisms
Source: Mob DNA. 2024 Jun 26;15:13. doi: 10.1186/s13100-024-00323-y (PMC11202377; doi:10.1186/s13100-024-00323-y)
Supplement: Supplementary file 1 — Supplementary Material 1. [file 13100_2024_323_MOESM1_ESM.pdf]

Supplementary Figures

Supplementary figure 1: Clustering strategy employed by pantera.

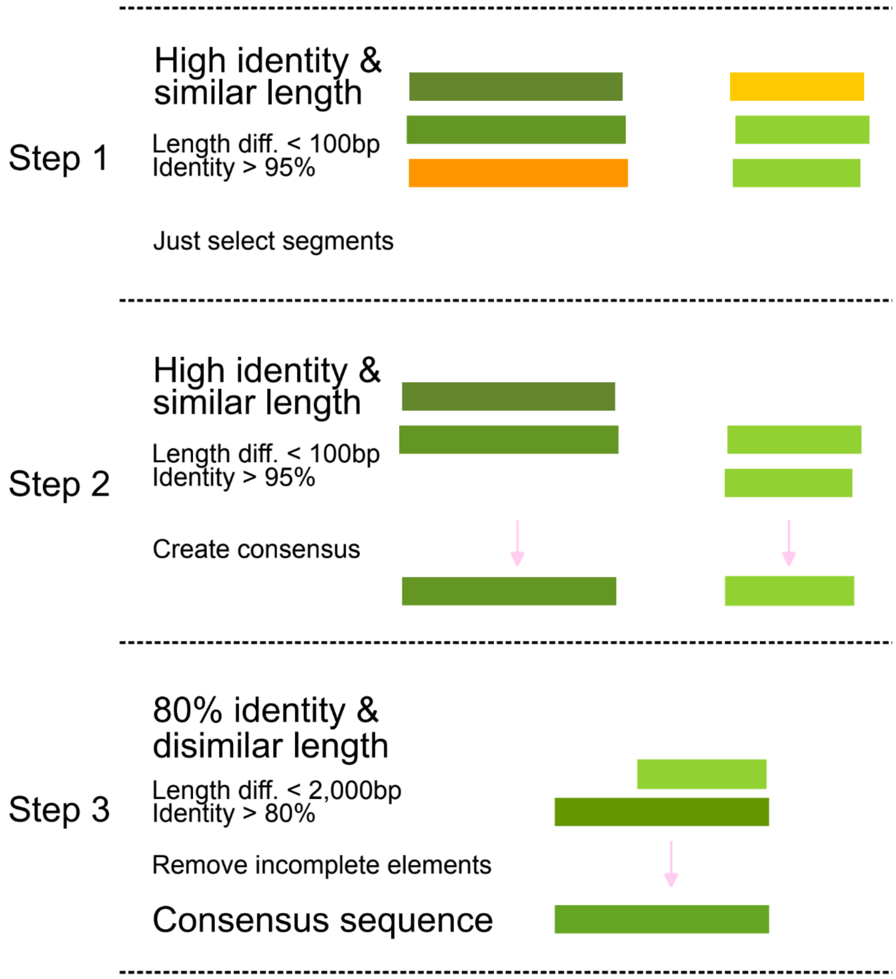

Clustering strategy applied by pantera. 1) Extract polymorphic segments from the pangenome of a certain size (defaults 250bp <= length <= 20,000bp). Divide them based on length into overlapping bins of width 200bp (100 overlapping bases, each sequence in two bins). Cluster them and select those that have at least one match of 95% identity. 2) Remove duplicate segments from Step 1 and cluster again with the same parameters. Create a plurality consensus of the sequences (default plurality threshold = 60%) 3) Collapse partial consensus sequences from Step 2 into complete sequences with max length difference = 2,000bp.

Supplementary figure 2: Length distributions of the different libraries by TE superfamily.

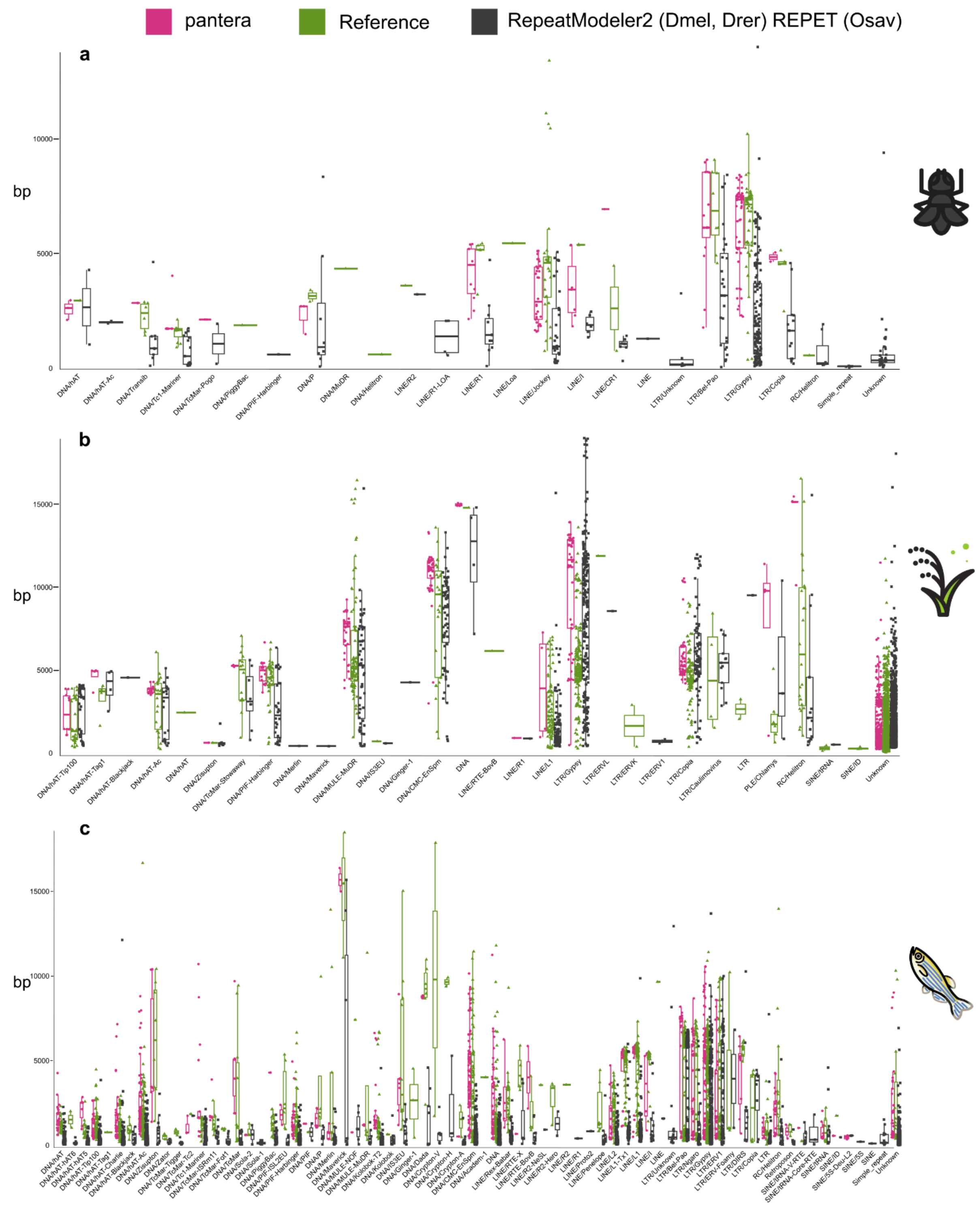

Length distributions of the consensus sequences by superfamily in which they have been classified. RC stands for rolling circle (Helitrons). **a***, Drosophila melanogaster.* pantera (N=141), Drosophila Transposon Canonical Sequences 10.2 (N=127), RepeatModeler (N=361). **b***, Oryza sativa.* pantera (N=525), rice6.9.5 (N=2431), REPET (N=2471) **c***, Danio rerio.* pantera (N=913), Dfam curated (N=1740), RepeatModeler (N=3728).

Supplementary figure 3: CMC-EnSpm family from *Danio rerio*.

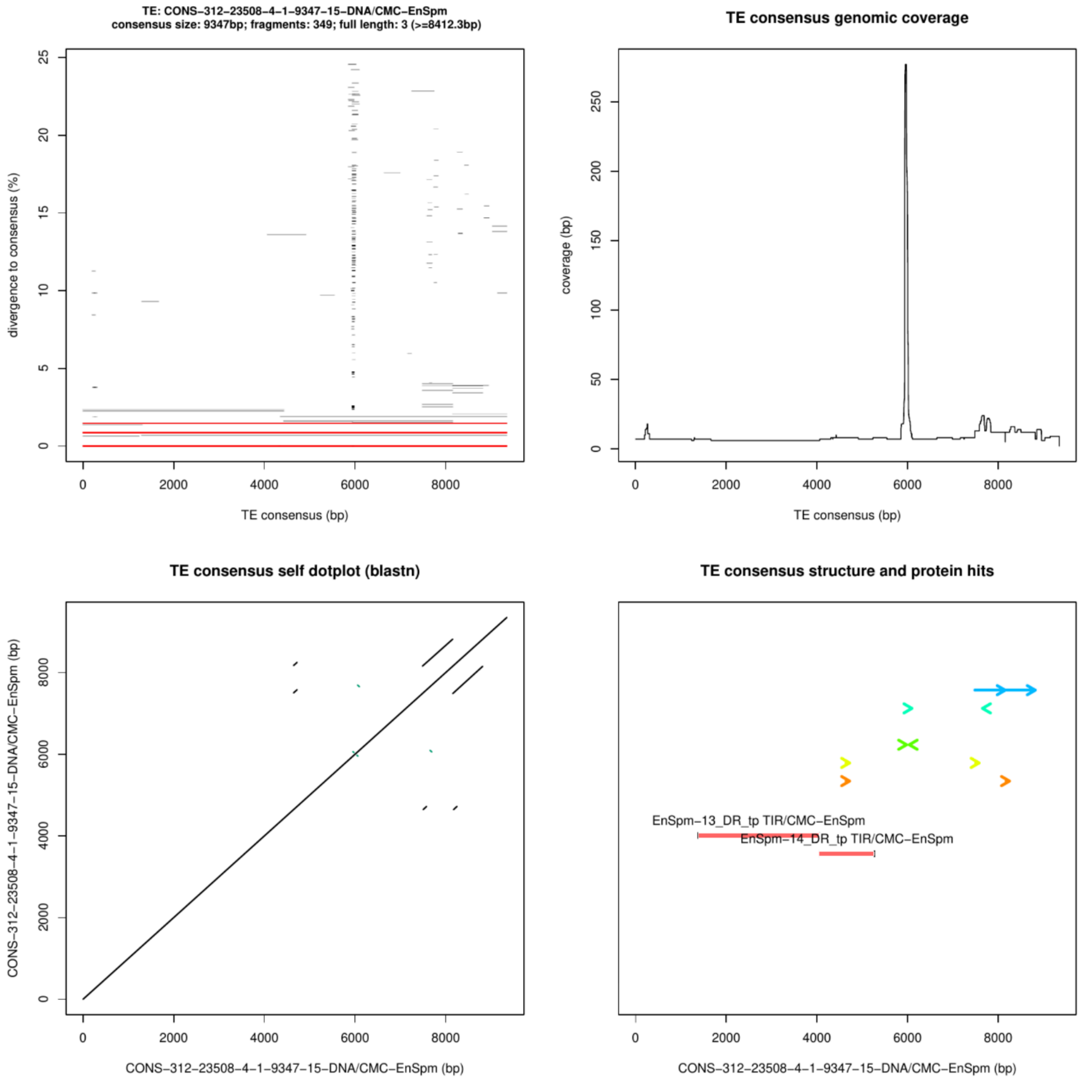

Plots obtained with TE-Aid for a CMC family found in *Danio rerio.* **a,** annotations in the genome by length and divergence to consensus. **b,** TE coverage of the annotations. **c,** TE consensus self dotplot. **d,** Structural features and proteins found in the TE.

Supplementary figure 4: Zisupton candidate family from *Danio rerio*.

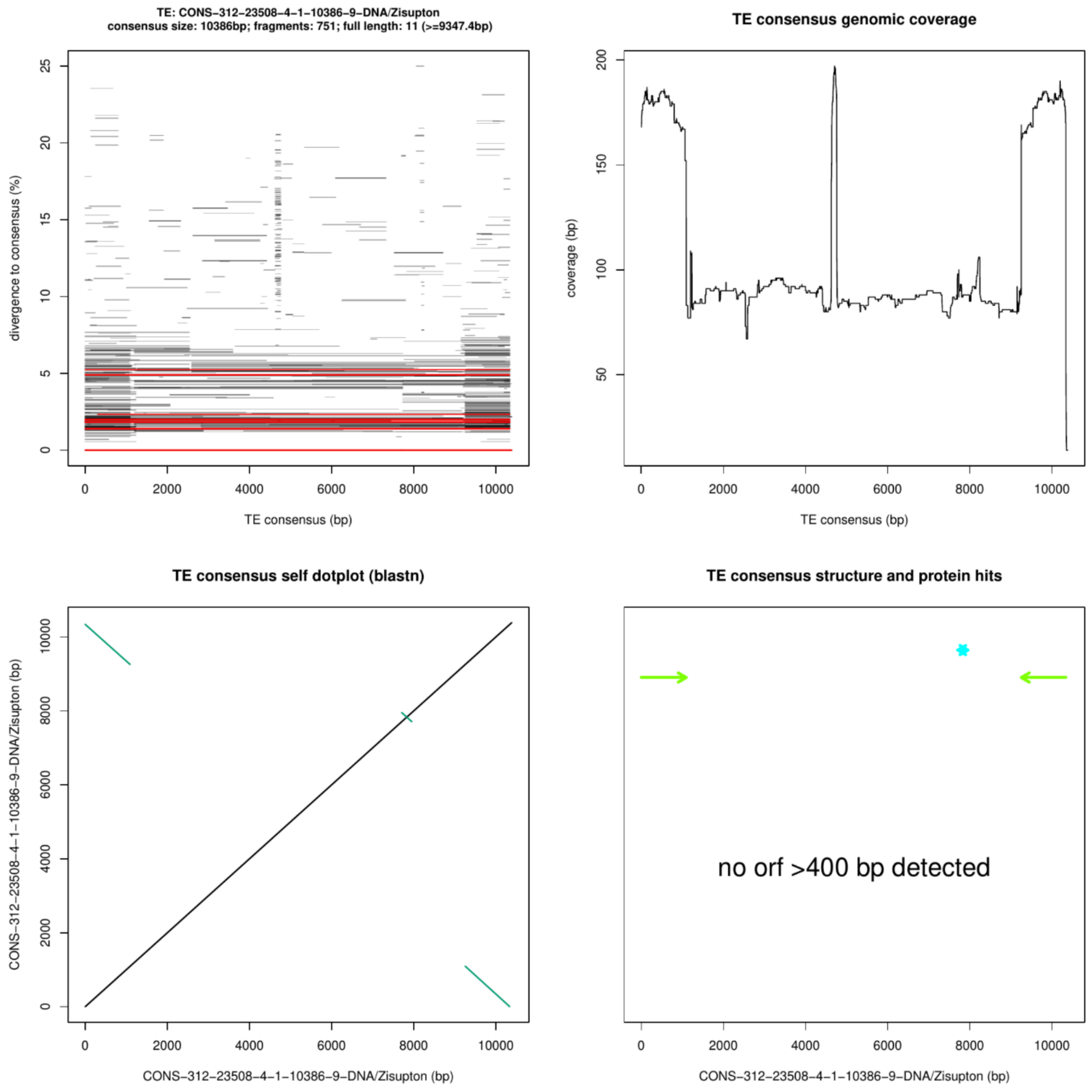

Plots obtained with TE-Aid for a new candidate Zisupton family found in *Danio rerio.* **a,** annotations in the genome by length and divergence to consensus. **b,** TE coverage of the annotations. **c,** TE consensus self dotplot. **d,** Structural features and proteins found in the TE.

Supplementary figure 5: RepeatMasker generated TE landscapes for *Drosophila melanogaster.*

a Reference library

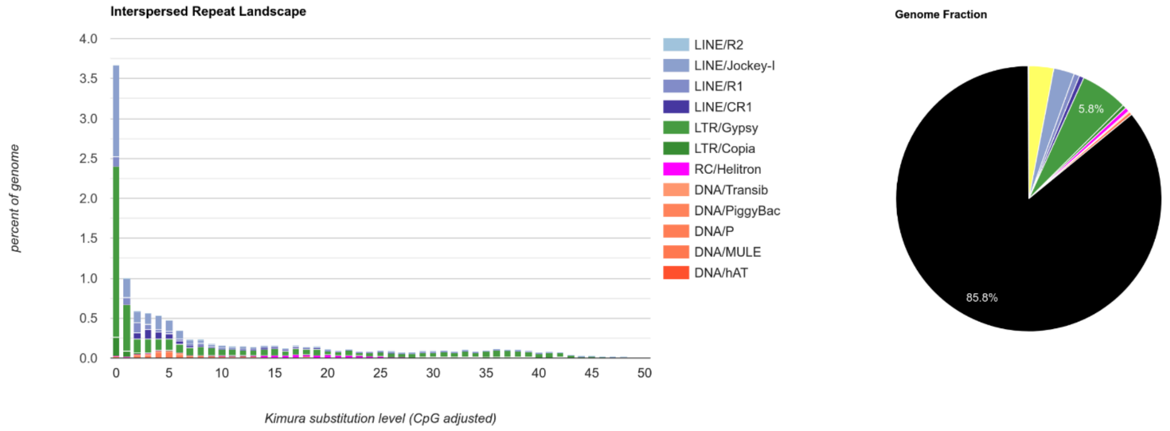

b pantera library

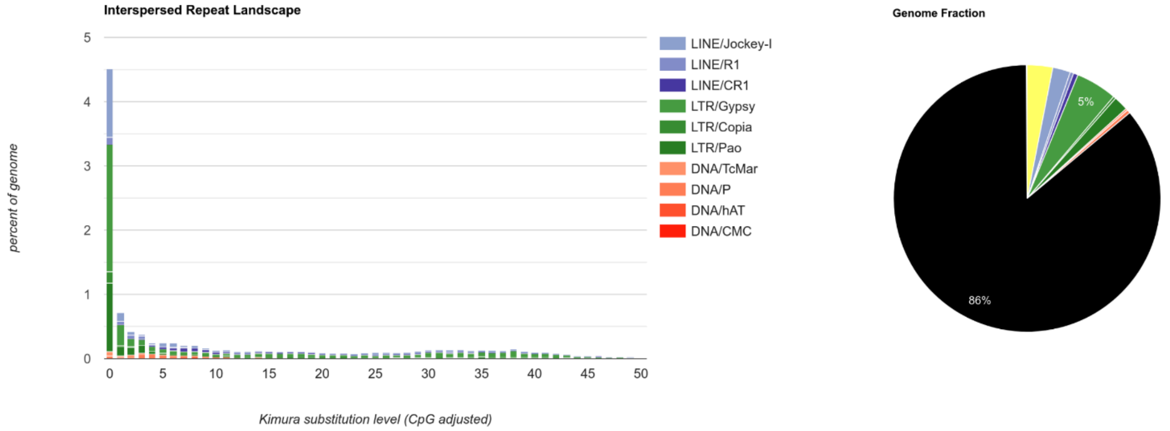

c RepeatModeler library

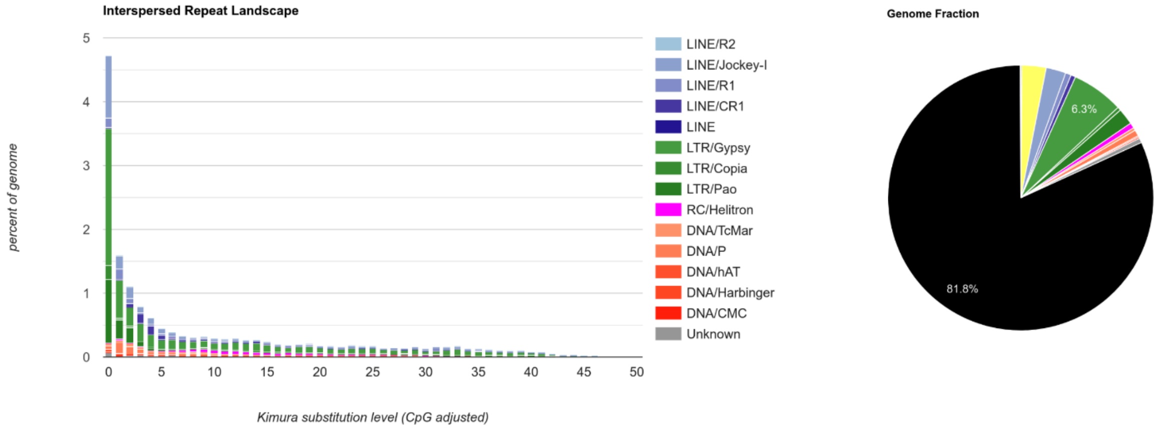

a, landscape obtained annotating the genome with the reference library. **b,** landscape obtained annotating the genome with the library obtained with pantera. **c,** landscape obtained annotating the genome with the library obtained with RepeatModeler.

Supplementary figure 6: RepeatMasker generated TE landscapes for *Oryza sativa.*

a Reference library

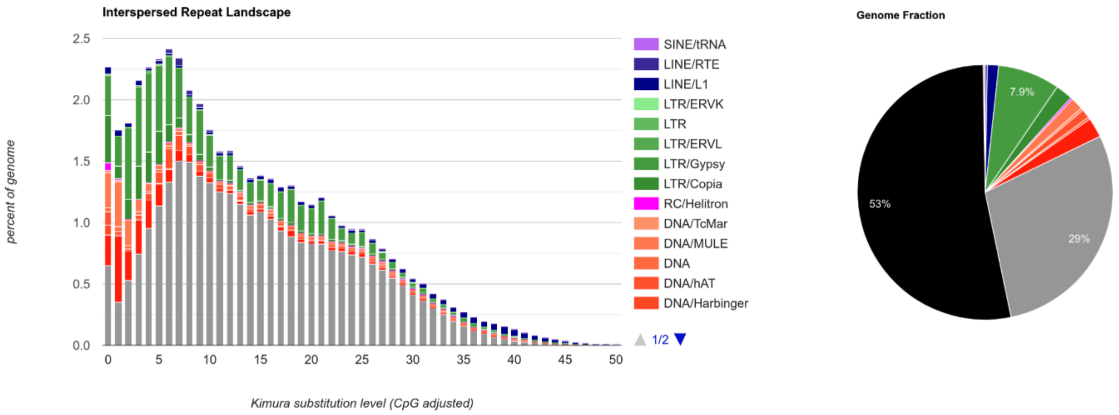

b pantera library

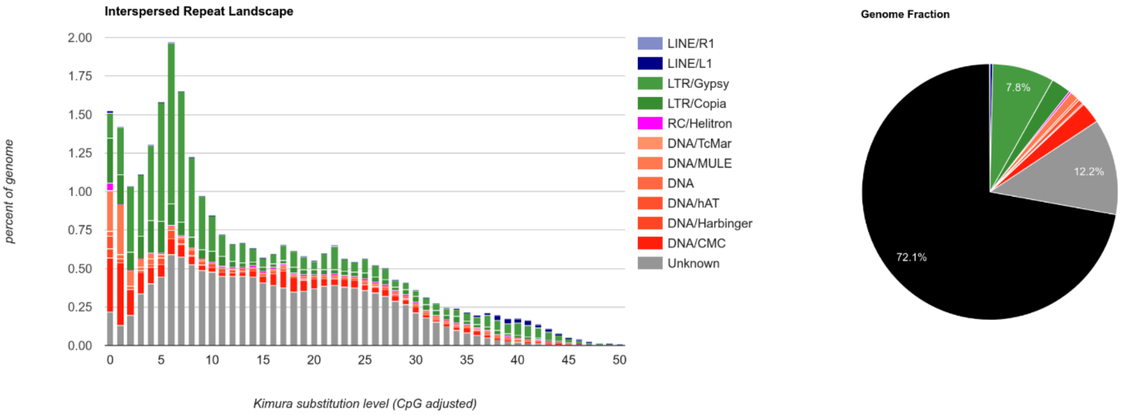

c REPET library

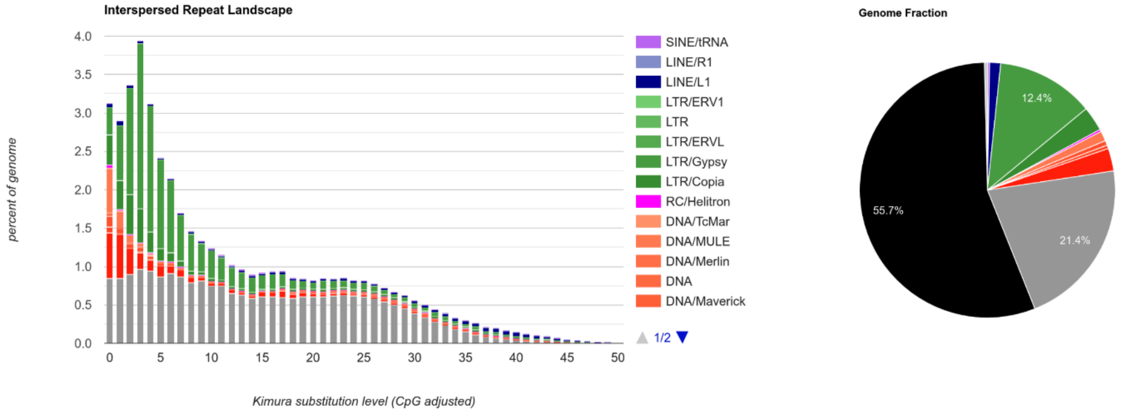

a, landscape obtained annotating the genome with the reference library. **b,** landscape obtained annotating the genome with the library obtained with pantera. **c,** landscape obtained annotating the genome with the library obtained with REPET.

Supplementary figure 7: RepeatMasker generated TE landscapes for *Danio rerio.*

a Reference library

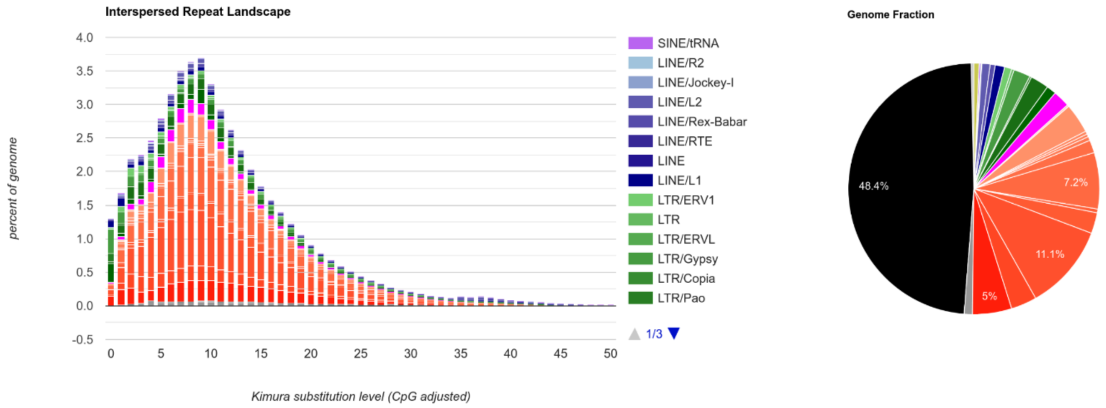

b pantera library

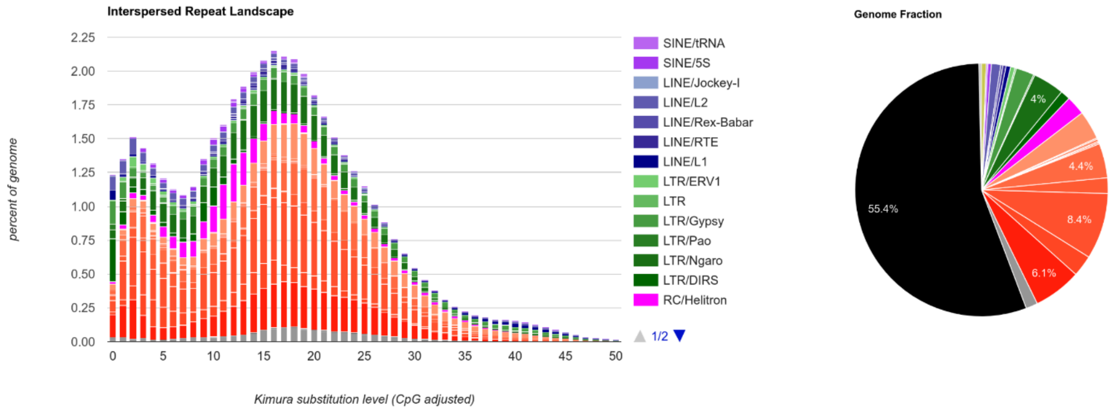

c RepeatModeler library

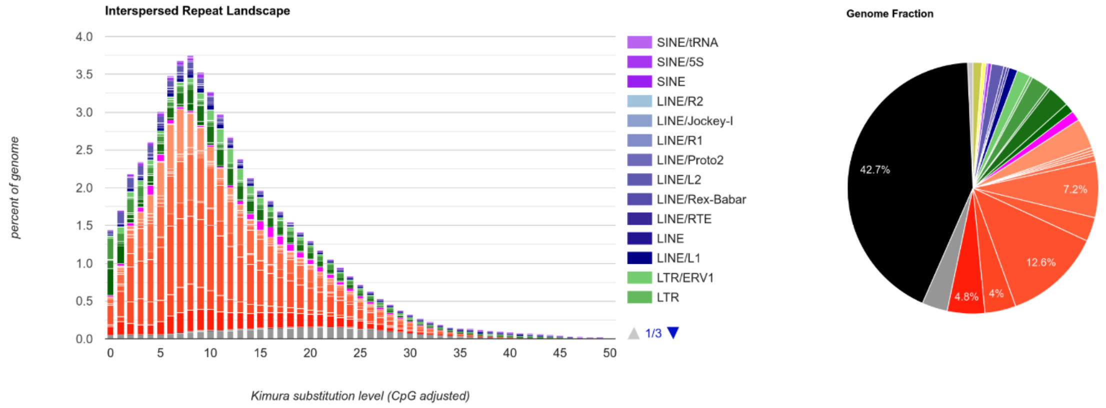

a, landscape obtained annotating the genome with the reference library. **b,** landscape obtained annotating the genome with the library obtained with pantera. **c,** landscape obtained annotating the genome with the library obtained with RepeatModeler.

Supplementary figure 8.: TE library construction times

Drosophila melanogaster	**Time**	**Threads**
Pangenome (pggb)	1h:32m	8
pantera	10m	8
RepeatClassifier	17m	8*
Total pantera workflow	1h:59m	
RepeatModeler	7h:28m	16
Oryza sativa*		
Pangenome (pggb)	2h:57m	16
pantera	1h:05m	16
RepeatClassifier	47m	8*
Total pantera workflow	4h:49m	
Danio rerio		
Pangenome (pggb)	6h:24m	24
pantera	1h:48m	24
RepeatClassifier	1h:04m	8*
Total pantera workflow	9h:16m	
RepeatModeler	61h:32m	32

Tests performed in the University of Cambridge HPC, with Dell PowerEdge XE8545 servers using 3rd Generation AMD EPYC 64-Core CPUs.

* RepeatClassifier has no multithreading support.

** No RepeatModeler2 run for Oryza sativa, an existing result from REPET was used instead.

Supplementary figure 9: Example of correct and incorrectly annotated TEs (large ERVs) in the TE libraries for *Aquila cryasetos*.

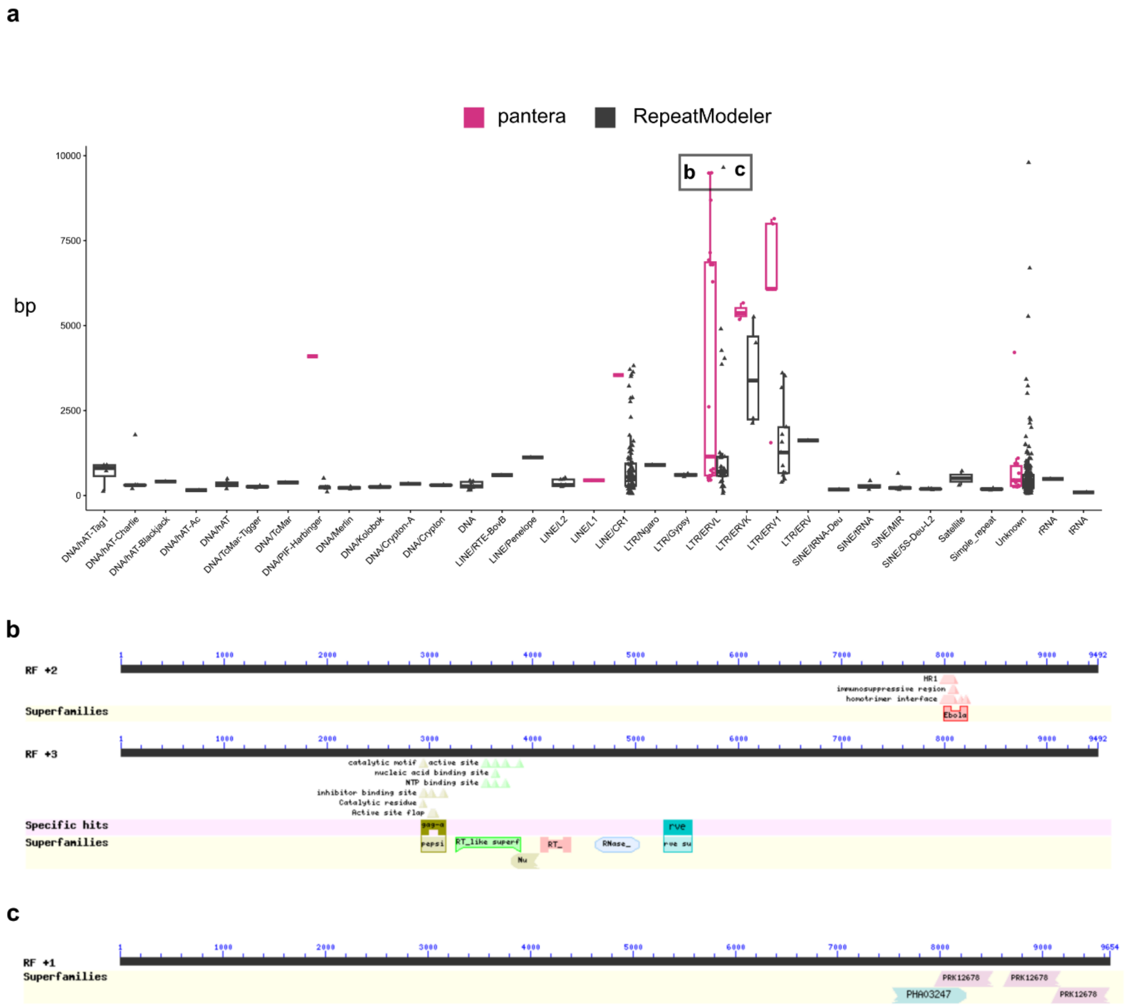

d

	Description	Scientific Name	Max Score	Total Score	Query Cover	E value	Per. Ident	Acc. Len	Accession
☑	uncharacterized protein LOC121233072 [Aquila chrysaetos chrysaetos]	Aquila chrysaetos chrysaetos	1019	1019	100%	0.0	98.62%	509	XP_040977606.1
☑	PREDICTED: uncharacterized protein LOC104828198 [Haliaeetus leucocephalus]	Haliaeetus leucocephalus	988	988	100%	0.0	95.68%	509	XP_010560005.1
☑	uncharacterized protein LOC126035454 [Accipiter gentilis]	Accipiter gentilis	986	986	100%	0.0	95.68%	509	XP_049650013.1
☑	uncharacterized protein LOC126034690 [Accipiter gentilis]	Accipiter gentilis	655	655	64%	0.0	97.87%	328	XP_049648616.1
☑	PREDICTED: uncharacterized protein LOC103924890 [Pygoscelis adeliae]	Pygoscelis adeliae	606	606	100%	0.0	63.38%	664	XP_009331774.1
☑	hypothetical protein N337_13015 [Phoenicopterus ruber ruber]	Phoenicopterus ruber ruber	534	534	87%	0.0	60.75%	444	KFQ77179.1
☑	uncharacterized protein LOC128136180 [Harpia harpyja]	Harpia harpyja	528	528	56%	0.0	94.43%	287	XP_052631320.1
☑	uncharacterized protein LOC127028179 [Gymnogyps californianus]	Gymnogyps californianus	514	514	98%	5e-175	57.22%	528	XP_050769900.1
☑	uncharacterized protein LOC127015429 [Gymnogyps californianus]	Gymnogyps californianus	508	508	98%	9e-173	56.38%	529	XP_050751272.1

a*,* Length distributions of the consensus sequences by superfamily in which they have been classified, highlighting the two largest ERV elements found by each tool. **b***,* Protein domains found in the pantera ERV sequences, which belong to the putative proteins of ERV elements. **c***,* Protein domains found in the RepeatModeler ERV sequence, the lack of ERV related proteins suggest it is either an artefact or the consensus has not been properly resolved. **d***,* The same ERV protein can be found in other species of the same clade. Images **b** and **c** obtained using the CD-Search service from NCBI. Image **d** obtained using the blastn service from NCBI.

Supplementary figure 10: LINE/L1 family from *Danio rerio*.

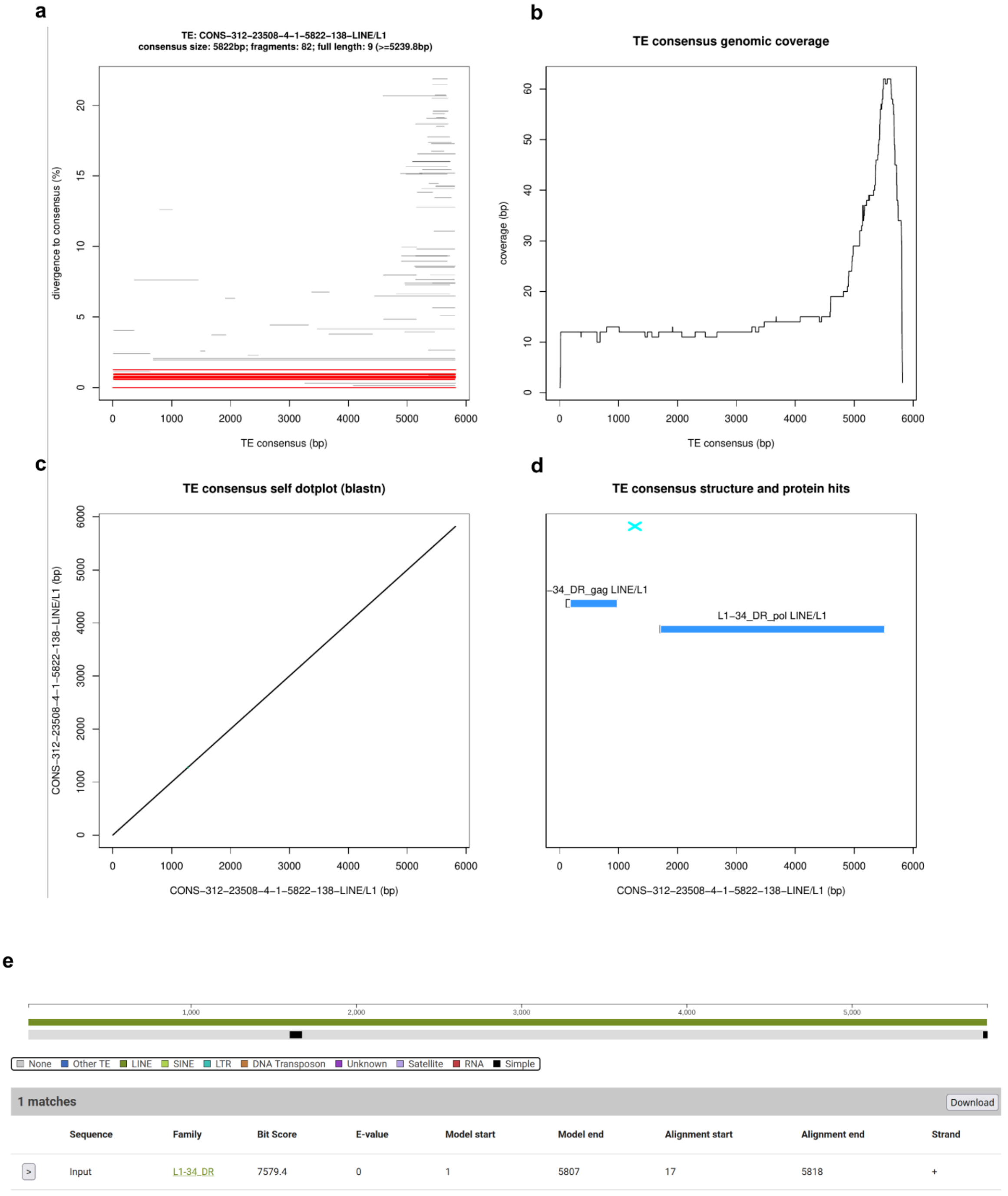

Plots obtained with TE-Aid for a LINE/L1 family found in *Danio rerio.* **a,** annotations in the genome by length and divergence to consensus. **b,** TE coverage of the annotations. **c,** TE

consensus self dotplot. **d,** Structural features and proteins found in the TE. **e,** identity of the LINE element with the current annotation in Dfam.
